# Negative Impacts of Psychiatric Medications on Oral Health: A Literature Review

**DOI:** 10.7759/cureus.49915

**Published:** 2023-12-04

**Authors:** Hassan Abed, Yousef Ezzat, Layan Alsaadawi, Rayan Almarzouki, Reema Aboulkhair, Ali Alqarni, Rayan Sharka

**Affiliations:** 1 Department of Basic and Clinical Oral Science, Umm Al-Qura University, Makkah, SAU; 2 Dentistry, Yanbu Specialized Dental Center, Riyadh, SAU; 3 Dentistry, Ibn Sina National College of Medical Sciences, Jeddah, SAU; 4 Department of Dental Services, King Abdulaziz Medical City, Jeddah, SAU; 5 College of Dentistry, King Abdulaziz University Dental Hospital, Jeddah, SAU; 6 Department of Oral and Maxillofacial Surgery and Diagnostic Sciences, University of Taif, Taif, SAU; 7 Department of Oral and Maxillofacial Surgery, Umm Al-Qura University, Makkah, SAU

**Keywords:** dementia, alzheimer, bipolar, antipsychotics, obsessive-compulsive disorder (ocd), schizophrenia, special care dentistry

## Abstract

Evidence reveals that people with mental illnesses have a greater risk of experiencing oral diseases such as dry mouth and dental caries and have greater oral treatment needs. This is related to multifactorial causes such as factors related to the condition itself, symptoms, side effects of polypharmacy, and a lack of accessibility to dental services. This article aims to provide a summary of the reported prevalence of the most common mental illnesses in Saudi Arabia (SA), such as schizophrenia, obsessive-compulsive disorder (OCD), bipolar disorders, and dementia. The article further aims to review the negative impacts of anti-psychotic medications on oral health and the role of dentists toward people with mental illnesses. PubMed, Scopus, and Google Scholar were searched using the following keywords: special care dentistry, schizophrenia, OCD, bipolar disorder, and dementia. The main inclusion criteria were any studies describing “dental management of patients with mental illnesses” and “dental management of patients treated with anti-psychotic medications.” Thematic analysis was used to summarize the findings of the included studies into main headings. Forty-eight articles/studies discussed dentistry, people with mental illnesses, and/or the negative impacts of psychotic medication on oral health. All studies were published between 1991 and 2021. In SA, the number of people with mental illnesses is increasing. Therefore, it is crucial for the dental team to understand the negative impacts of anti-psychotic medications on oral health, such as dry mouth and the increased risk of dental caries. This necessitates the need for regular dental visits and patient education on how they can mitigate the side effects of anti-psychotic medications on oral and general health.

## Introduction and background

The special populations of adults, elderly people, and pregnant women with mental health illnesses have unique requirements and require enhanced care services in different settings [1]. The World Health Organization (WHO) estimated that one in four people in the world's population is diagnosed with mental health illness in developed and developing countries [2]. The Ministry of Health (MOH) in Saudi Arabia (SA) is the main provider of public mental health services. Under its umbrella, the General Administration for Mental Health and Social Services plans, implements, coordinates, evaluates, and monitors mental health service delivery and also follows the core themes of the WHO in developing mental health services. In 2015, a focusing review of the literature indicated the lack of an accurate estimate for the prevalence of mental health illness in the SA [3]. Three years later, a cross-sectional study found that the prevalence of mental illness in the capital of SA was 28.5% [4].

To our knowledge and based on the literature findings, there are 21 mental health hospitals established in SA to meet the needs of psychiatric patients [5]. In the near future, this will increase to 27 mental hospitals, as there are six projects under development with an increase of seven hospitals over a ten-year period [5]. Eight of these mental hospitals integrated their facilities for the purposes of prevention, treatment, and research involvement [5]. Stigma and discrimination against people suffering from mental illnesses have a negative impact on their education, employment, and access to care, as well as their ability to contribute to society [6]. People with mental illnesses have their rights compared to the general population, autonomy, and equal medical and dental treatment standards. Therefore, mental health policies should try to improve access to all services [7]. For example, increasing awareness among medical and dental care providers about basic knowledge of mental illness will help reduce stigma toward the management of people with mental illness. Besides, improving the accessibility of people with mental illness through easy access, regular medical follow-up, and psychological support will help them receive better and safer medical and dental care [8]. A Saudi human rights committee is responsible for assessing the quality of mental health facilities and imposing sanctions on facilities that persistently violate patients’ rights [9]. In SA, oral health status is worse among patients with psychiatric and mental health illnesses, who are more likely to develop some oral conditions, such as temporomandibular disorders, dry mouth, and dental caries [10]. Additionally, evidence reveals that they have a greater risk of experiencing oral disease and have greater oral treatment needs [11,12]. This is related to multifactorial causes such as factors related to the condition itself, symptoms (i.e., difficulty maintaining good oral hygiene), side effects of the polypharmacy (i.e., dry mouth-related dental caries), and indeed, lack of accessibility to dental services (i.e., lack of well-trained dentists) [11,12].

Therefore, it is crucial for dental and mental healthcare providers to be aware of patients' needs and the preventive measures to be instituted for them. This article aims to provide a summary of the reported prevalence of the most common mental illnesses in SA, such as schizophrenia, obsessive-compulsive disorder (OCD), bipolar disorders, and dementia. The article further aims to review the negative impacts of anti-psychotic medications on oral health and the role of dentists toward people with mental illnesses.

## Review

Methodology

**Figure 1:**
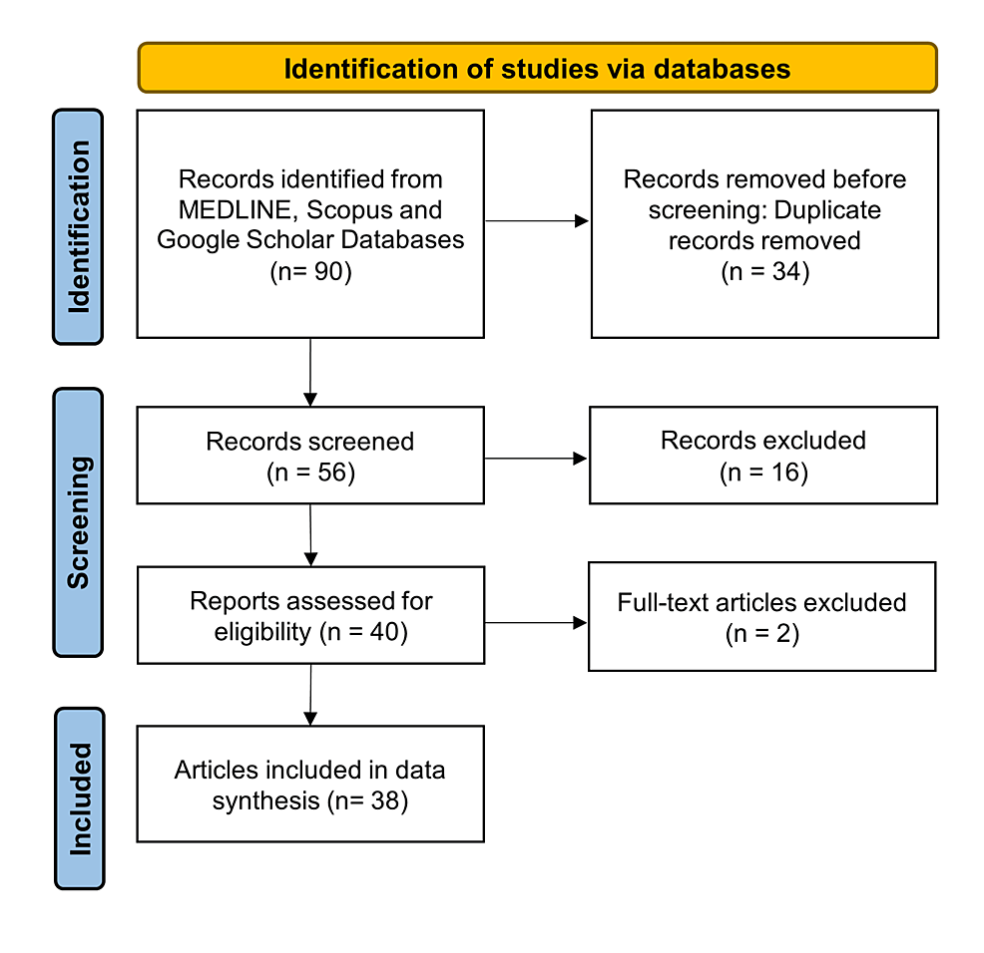
Flow diagram of the selection process

This review was developed as per PRISMA guidelines (see Figure 1). The review aimed to answer the following questions: what is the prevalence of the most common mental illnesses in SA? and what are the negative impacts of anti-psychotic medications on oral health?

Eligibility Criteria

All types of articles in English published from 1991 to 2021 were included during the search process. However, conference posters, letters to the editor, expert comments, and partial texts were not included in this study.

Exclusion Criteria

Articles that were published utilizing a qualitative approach, published prior to the mentioned period, and published in a language other than English were excluded. Also, studies that did not include details about mental illnesses other than the ones selected for this review, such as "schizophrenia,” “obsessive-compulsive disorder (OCD)," “bipolar disorder," and "dementia," were excluded. Moreover, case reports, case series, letters to editors, conference papers, and unpublished studies were excluded. Also, studies that included participants aged less than 18 years old were excluded.

Search Strategy

Ovid MEDLINE, Scopus, and Google Scholar databases were considered to retrieve eligible studies using planned search keywords such as “Mental illness in Saudi Arabia,” “impact of anti-psychotic medications,” “Special care dentistry,” “schizophrenia,” “obsessive-compulsive disorder (OCD)," “bipolar disorder,” and “dementia.”

Study Selection and Data Analysis 

Titles, abstracts, and keywords of the possible studies were screened by two evaluators (HA and RS). Included articles were summarized into thematic headings: schizophrenia, OCD, bipolar disorder, dementia, general dental considerations, the stigma of treating people with mental illness, and accessibility of people with mental illness to dental services.

Results

Thematic analysis was used to summarize the findings of the included studies into main headings. This article included 48 articles/studies that discussed dentistry and people with mental illnesses and/or the negative impacts of psychotic medication on oral health. All studies were published between 1991 and 2021. A summary of the basic details about mental illnesses and the negative impact of anti-psychotic medications on oral and dental health are discussed below. 

Schizophrenia

Although schizophrenia has been considered a unique disease in the last decades, its definitions, types, and etiology have frequently changed. Frith (2014) defined schizophrenia in a simple manner as a “psychotic disorder that may compromise a group of disorders” [13].

In SA, the incidence of schizophrenia is surprisingly scarce, with limitations of the research and published scientific articles assessing the schizophrenia-related aspects. For instance, in 2008, the MOH in SA reported that 22.4% of outpatients in mental health services had a mental condition that was caused by schizophrenia [14]. However, this percentage increased recently. For example, a large, cross-sectional study in SA psychiatric settings found that schizophrenia affected 55.8% of inpatients (Total=443) and 28.9% of outpatients (Total=762) [15].

Psychiatrists tend to label schizophrenia with positive and negative signs and symptoms based on the patient’s behavior [13]. For example, hallucinations and delusions are considered positive signs and symptoms, while loss of energy, less interest in life, and negative thoughts are considered negative signs and symptoms [16]. The later signs and symptoms compromise the patient’s engagement with the psychiatrist's medical and psychological care [16]. Hallucination includes different behavioral forms such as visual, olfactory, somatic, and gustatory with the most common form of auditory hallucination in both types: verbal and non-verbal [13]. Delusion is a false fixed belief that may dominate the person's mind such as feeling persecutory, powerful, or dyeing of one of the body’s organs [17].

Currently, many studies try to find out the exact reason for schizophrenia but it remains unclear [18]. Evidently, it is popularly known as a multifactorial disease. For instance, genetic predilection between the first-degree relative, structural brain abnormalities, neurotransmitter deficiency, psychoactive substances, and pre-natal complications such as bleeding during the pregnancy and late winter birth [14].

Medical management is usually started through psychological intervention and variant types of typical and atypical anti-psychotic medications, which clearly show a negative impact on oral health and affect dental treatment delivery. The most common side effects of variant types of anti-psychotic medications are discussed in this article alongside actions that can be taken by dentists during dental treatment to help deliver proper oral health care (see Table 1).

**Table 1:** Adverse effects of typical and atypical anti-psychotic medications with dental recommendations

Drug type		Side effects	Dental recommendations
Typical anti-psychiatric medications	Chlorpromazine	Extra-pyramidal effects: Parkinsonian symptoms, dry mouth, nasal congestion, blurred vision, involuntary facial movement, akathisia, stress and restlessness, postural hypotension, neuroleptic malignant syndrome, and cardiac dysrhythmias	Patients are characterized by slow body movement; consider easy access to the dental chair and adjust the headrest position with a pillow. A dry mouth increases the risk of dental caries, candidiasis, gingivitis, periodontitis, and difficult speech. It is suggested to advise patients to drink lots of water and use high-fluoride toothpaste (5000 ppm) with regular dental check-ups. Agitation and depression might affect the patient’s decision for dental treatment. It is suggested to postpone the irreversible treatment, such as dental extraction, when the patient is in a good mood to decide and consider easily reversible treatments, such as dental restorations and scaling. Dental care providers should be aware of postural hypotension and adjust the dental chair slowly. Check white blood cells for “agranulocytosis” prior to dental extraction to reduce the possibility of infection, especially in patients with typical psychotic medications. Consider conscious sedation such as nitrous oxide to help reduce dental anxiety levels, control involuntary muscle movement, and help deliver proper dentistry. Dental care providers should be aware of possible drug interactions between anti-psychotic medications and medications usually prescribed in dental practice.
	Haloperidol		
	Fluphenazine		
	Flupentixol		
	Trifluoperazine		
	Pimozide		
Atypical anti- psychiatric medications	Clozapine	Hypersalivation, sedation, weight gain, agranulocytosis, convulsion, and long-term use might reduce hepatic function	
	Risperidone	Hyperprolactinemia, impaired concentration, anxiety, fatigue, gynecomastia, and weight gain	
	Olanzapine	Blood dyscrasias, dizziness, and sedation effect	
	Quetiapine	Dry mouth, stress, sedation effect, and reduced white blood cells	

Obsessive-Compulsive Disorder

Another example of a common mental illness is an obsession thought that causes anxiety, which leads to repetitive actions that are carried out compulsively to help eliminate established anxiety [19]. It affects a person in carrying out daily activities. Altered serotonin transporter binding shows relative reasons [20]. Additionally, genetic contribution and personality type might increase the possibility of developing OCD [21,22]. In SA, the prevalence of OCD was reported to be 3.4% to 23.1% [23-25]. Another cross-sectional study found that OCD affected 1.1% of inpatients (Total=443) and 3.3% of outpatients (Total=762) [26]. The difference in the prevalence of OCD in SA is affected by the regions in which SA studies are conducted and the differences in tools used to assess the prevalence of OCD. Two cross-sectional studies reported that OCD was higher among adolescent single females than males [23,27].

Diagnosis of OCD is not an easy task, as it requires patient self-reporting and frequent behavioral observation. Selective serotonin reuptake inhibitors (SSRIs) are antidepressant medications used in the treatment of OCD patients. Table 2 shows the most common SSRIs prescribed and their side effects on oral and general health.

**Table 2:** The most common SSRIs prescribed and their side effects on oral and general health SSRIs, selective serotonin reuptake inhibitors

Medications	Side effects on oral health	General side effects
Paxil (paroxetine)	Dry mouth and dry oropharynx affecting oral functioning, such as eating and speech. Cracked lips, multiple oral ulcers, oral burning sensation, taste disturbance, bruxism, bad breath	Abdominal disturbance, blurred vision, weight instability, high blood glucose level, increased anxiety, and anger. Feeling unwell, increased sweating glands production, sexual dysfunction
Zoloft (sertraline)		
Lexapro (escitalopram)		
Celexa (citalopram)		
Prozac (fluoxetine)		

Fluoxetine (Prozac®) is a common SSRIs used in the treatment of patients with OCD [28]. Fluoxetine has side effects such as nausea, diarrhea, headache, jitteriness, insomnia, fatigue, and sexual dysfunction [29]. Fatigue can affect patients’ attendance at the dental clinic and swing their moods. Nausea in some cases can lead to frequent vomiting, which is sometimes why patients present with chemical erosion for the enamel surface. Frequent diarrhea can lead to dry mouth due to reduction of the body’s electrolytes; hence patients are susceptible to dental caries. Sudden withdrawal from using fluoxetine can lead to dizziness, dysphoria, and severe anxiety, which sometimes requires special care dentistry through using conscious sedation. In addition to SSRIs, cognitive behavioral therapy (CBT) shows effective results in managing patients with OCD [28,30].

Dental care providers can help to refer undiagnosed cases and seek an early opinion from the psychiatric team. For example, patients who brush their teeth vigorously and frequently without limited numbers with signs of multiple abrasion areas raise the suspicion of undiagnosed OCD, which requires discussion with the patients and liaison with the medical team.

Bipolar Disorder

Bipolar disorder is known as the presence of one or two abnormally elevated moods, clinically defined as a mania episode [31]. It established sole or adjunctive with the depression episode called “rapid cycling” [32]. Bipolar disorder is a multifactorial disease affected by biological, environmental, and genetic factors.

A large, cross-sectional study in SA psychiatric settings found that bipolar disorder affected 23.3% of inpatients (Total=443) and 11.5% of outpatients (Total=762) [26].

The main goal of the treatment of bipolar disorder is to prevent and stabilize the condition [33]. For example, psychological intervention with CBT is a valuable technique used to manage patients diagnosed with bipolar disorders [34]. Adjunctive mood stabilizer (i.e., lithium) and anticonvulsant (i.e., sodium valproate) agents could be used [35,36]. Adjunctive mood stabilizer agents affect negatively maintaining oral health care as they produce xerostomia and gingival enlargement, which increase the risk of dental caries and complicate maintaining good oral hygiene [37].

Providing simple education for the patients is considered a part of the treatment regimen. Smoking cessation programs and the reduction of alcohol drinks are also valuable in managing patients with bipolar disorder to reduce risk to oral health [38,39].

Dementia

Dementia is defined as a chronic progressive mental disorder that adversely affects higher cortical functions including memory, thinking, orientation, comprehension, calculation, learning capacity, language, and judgment [40]. It affects the retrieval, storage, and registration of new information [41].

WHO reported the importance of the financial budget in the mental health sector “mental health financing is a powerful tool with which policy-makers can develop and shape quality MHSs. Without adequate financing, mental health policies and plans remain in the realm of rhetoric and good intentions” [42]. In 2009, the total estimated worldwide costs of dementia were US$422 billion [43]. This cost had been increased in 2010 to US$604 [44]. However, worldwide costs of dementia are enormous and distributed inequitably [43]. In 2013, 4% of the entire healthcare budget of the MOH was directed toward mental health care [9]. In 2015, the budget of MOH has been increased to 62.34 billion. However, it is not easy to estimate the budget for the mental health trend in SA as mental health financing needs further support from the Saudi government [5,9]. Unfortunately, while waiting for an accurate budget, the number of patients with mental health will rise. 

A large, cross-sectional study in SA psychiatric settings found that dementia affected 0.5% of inpatients (Total=443) and 2% of outpatients (Total=762) [26]. It is a massive future challenge that faces SA, as the number of Saudi people with dementia is increasing [25].

Dementia is not affecting solely elderly people; it can affect all age groups [45]. The National Institute for Health and Excellence (NICE) suggested that a person is diagnosed with dementia if there is a sign of memory loss for more than 12 months [46]. However, diagnosis of dementia is not an easy task, as it requires different medical and laboratory investigations. Five known types of dementia have been recognized, and these are Alzheimer's disease, vascular dementia, mixed dementia, Lew-body dementia, and frontal-temporal dementia [41]. These types have three progressive stages which are mild, middle, and late stages [45].

For example, patients diagnosed with early-stage dementia could provide signs for a dental team such as forgetting the last talk discussion, asking the same question at the same time repeatedly, can not remember the names of their family members, and cannot remember how many times or when the last time they brushed their teeth. In the moderate stage, patients usually rely on one of their family members and become independent in dressing, personal hygiene, brushing their teeth, and most of their day-to-day activities. Later on in the late stage, patients have difficulties accessing dental clinics as they might become wheelchair recliners or confined to bed, and they will find it difficult to accept or to receive dental treatment [45].

There are two main types of medications recommended by NICE for the treatment of Alzheimer’s disease, which are acetyl-cholinesterase inhibitors for mild-moderate conditions and NMDA receptor antagonists for severe forms of the disease [40]. Cholinesterase inhibitors such as donepezil hydrochloride (Aricept®), rivastigmine (Exelon®), and galantamine (Reminyl®) prevent an enzyme called “acetyl-cholinesterase” from breaking down acetylcholine in the nervous system. By contrast, the NMDA receptor antagonist such as memantine (Ebixa®) blocks chemical substances called “glutamate” and protects the brain from further damage. The side effects include loss of appetite, nausea, vomiting, diarrhea, stomach cramps, headaches, increased blood pressure, and fatigue [40].

Discussion

The Stigma of Treating People With Mental Health Illnesses

Stigma and discrimination against people living with mental illnesses affect their education, employment, and access to care and hamper their capacity to contribute to society. Community Mental Health Care (CMHC) helps to reduce stigma and deliver equal care for patients diagnosed with chronic mental illnesses [6]. However, it still required more improvements, as it could not provide services for patients with intellectual and developmental disabilities [47]. The deficient distribution of these community-based centers in SA is considered one of the main reasons for increasing the number of people with mental illnesses [5]. A national survey for the exact epidemiological oral health needs in the CMHC, primary health care, private sectors, and general hospitals in SA is a key factor in starting to establish oral and dental health services for this group in SA.

Improving Dental Accessibility for People With Mental Health Illnesses

People with mental health illnesses have difficult access to dental clinics. At present, the current bed occupancy is more than 100% (4805 beds in 21 hospitals) in SA [5]. Although there is an assigned dentist at each mental health hospital, this is not enough, as the number of people with mental illness who live at home and who visit primary and secondary dental clinics is not small. Besides, the British Society of Disability and Oral Health (BSDH) recommended providing oral health care for patients with mental health illnesses in different ways, such as domiciliary dentistry and mobile dental units [48,49]. Also, oral and maxillofacial clinics in psychiatric hospitals could be used for providing emergency dental treatment. Cost, fear, anxiety, and dental phobia are among the most common barriers to dental care for those patients [11,12]. Physical or mental illness may progress to develop self-neglection with the least priority for oral health care. Educate the patients to be independent with support such as maintaining self-oral care and easy access to available dental services. Continued educational programs for dental and mental health services are crucial to understand the available dental services to access, the way of referral, providing training courses to manage patients with mental health illnesses, and building up a smooth collaboration between both services.

The limitations of this review were that the quality assessment of the included articles was not conducted. It is important for future research to take this into consideration. Ensuring the comprehensive inclusion of all literature pertaining to mental illness is unattainable, so there is a chance that few studies were included in this review; hence, this limits our findings. However, the current review has a number of strengths. It included all studies that discussed the prevalence of mental illnesses in SA. Broad keywords were considered to search for eligible studies. Searching in PROSPERO, the database of systematic reviews, found that the current review is the first to aim to assess the prevalence of mental illnesses in SA. Also, two evaluators were independently involved in all stages of this review.

## Conclusions

Limited and robust studies are available that discuss the prevalence of mental illnesses in SA. Accordingly, the prevalence of mental illnesses as reported in the included studies of this review ranged from 22% to 55%. Therefore, it is crucial for the dental team to understand the negative impacts of anti-psychotic medications on oral health, such as dry mouth, and the increased risk of dental caries. This necessitates the need for regular dental visits and patient education on how they can mitigate the side effects of anti-psychotic medications on oral and general health.

Assessing the unmet oral health care needs of people with mental health illnesses requires the integration of dental and mental health services in primary, secondary, and tertiary care to provide an equal standard of dental treatment compared to the general population. Indeed, oral and mental health are essential elements underlying the improvement of the mental health system, which is required across SA.
